# Pulmonary tularaemia: all that looks like cancer is not necessarily cancer – case report of four consecutive cases

**DOI:** 10.1186/s12890-015-0026-y

**Published:** 2015-03-26

**Authors:** Patrick Fachinger, Gabrielo Mauro Tini, Rainer Grobholz, Franco Gambazzi, Hans Fankhauser, Sarosh Irani

**Affiliations:** Clinic of Pulmonary and Sleep Medicine, Cantonal Hospital Aarau, Tellstrasse, CH-5001 Aarau, Switzerland; Department of Pathology, Cantonal Hospital Aarau, Tellstrasse, CH-5001 Aarau, Switzerland; Clinic of Thoracic surgery, Cantonal Hospital Aarau, Tellstrasse, CH-5001 Aarau, Switzerland; Institute for Microbiology, Cantonal Hospital Aarau, Tellstrasse, CH-5001 Aarau, Switzerland

**Keywords:** Pulmonary tularaemia, FDG-PET-CT-scan, Endobronchial ultrasonography

## Abstract

**Background:**

Pulmonary tularaemia is a very rare disease with only a small number of cases described in the literature. So far, to our knowledge, there exists no case report of pulmonary tularaemia where PET-CT scans and follow up CT scans are available.

**Case presentation:**

We present four consecutive cases of pulmonary tularaemia. All patients suffered from non-specific symptoms. All patients were referred to our institution with strong suspicions of malignancy, particularly lung cancer.

Diagnosis of tularaemia was made by typical findings in the aspirate of EBUS guided fine needle aspiration (necrosis, epithelioid cell aggregation) and surgical biopsy respectively, and a positive serology. In three of the four cases, the diagnosis was confirmed by positive PCR results of the tissue. PET-CT scans obtained in all four cases were indistinguishable from lesions typically seen in patients suffering from lung cancer.

One of the four patients suffered from recurrence of the disease after antibiotic treatment; also this patient finally recovered after initiation of a second antibiotic regimen. One case became asymptomatic spontaneously, but this patient still received an antibiotic treatment.

In one case, a follow up CT scan was unchanged compared to the initial PET-CT scan; in all other cases, the lesions disappeared almost completely.

**Conclusions:**

Symptoms of patients suffering from pulmonary tularaemia are non-specific and can be of prolonged character. PET-CT scans in these cases are indistinguishable from lung cancer. The diagnosis can be established when typical findings in EBUS guided fine needle aspirates or surgical biopsies are found in combination with a positive serology. In most cases the lesions disappear in follow up CT scans after clinically successful treatment.

## Background

Due to their frequent occurrence, pulmonary malignancies are sometimes considered spot diagnoses. This is particularly true when modern techniques like positron emission tomography CT fusion scans (FDG-PET-CT-scans) are applied. Due to their high sensitivity and specificity, characteristic images and patterns can easily mislead to premature diagnosis of malignancy.

Pulmonary tularaemia on the other hand is a very rare disease with only a limited number of case reports and case series existing in the literature. Tularaemia is a rare zoonosis caused by Francisella tularensis. Different clinical manifestations such as oropharyngeal type, ulceroglandular type, oculoglandular type or typhoidal type have been reported. Pulmonary involvement occurs as a distinct pattern or association with other forms of the disease. In the literature, the frequency of pulmonary involvement varies substantially. In a review of 22 cases from Arkansas suffering from typhoidal tularaemia, 83% showed pulmonary involvement [1]. In a large cohort report of a tularaemia outbreak in Bulgaria, only nine (3.4%) out of 262 affected patients clinically showed pulmonary tularaemia [2]. Respiratory tularaemia may manifest with dry cough, chest pain, fever and dyspnea. The radiological presentation, in addition, varies markedly in the different case series available. Whereas lobar or multi-lobar infiltrates seem to be the manifestation most frequently reported [1,3,4], pleural effusions [4] and hilar adenopathy have also been observed [5]. To our knowledge, cases where FDG-PET-CT scans are available have not yet been described.

We present four consecutive cases with pulmonary tularaemia which have been treated at our institution and of which PET-CT scans and follow up CT scans are presented.

## Case presentation

### Patient No 1

**Figure 1:**
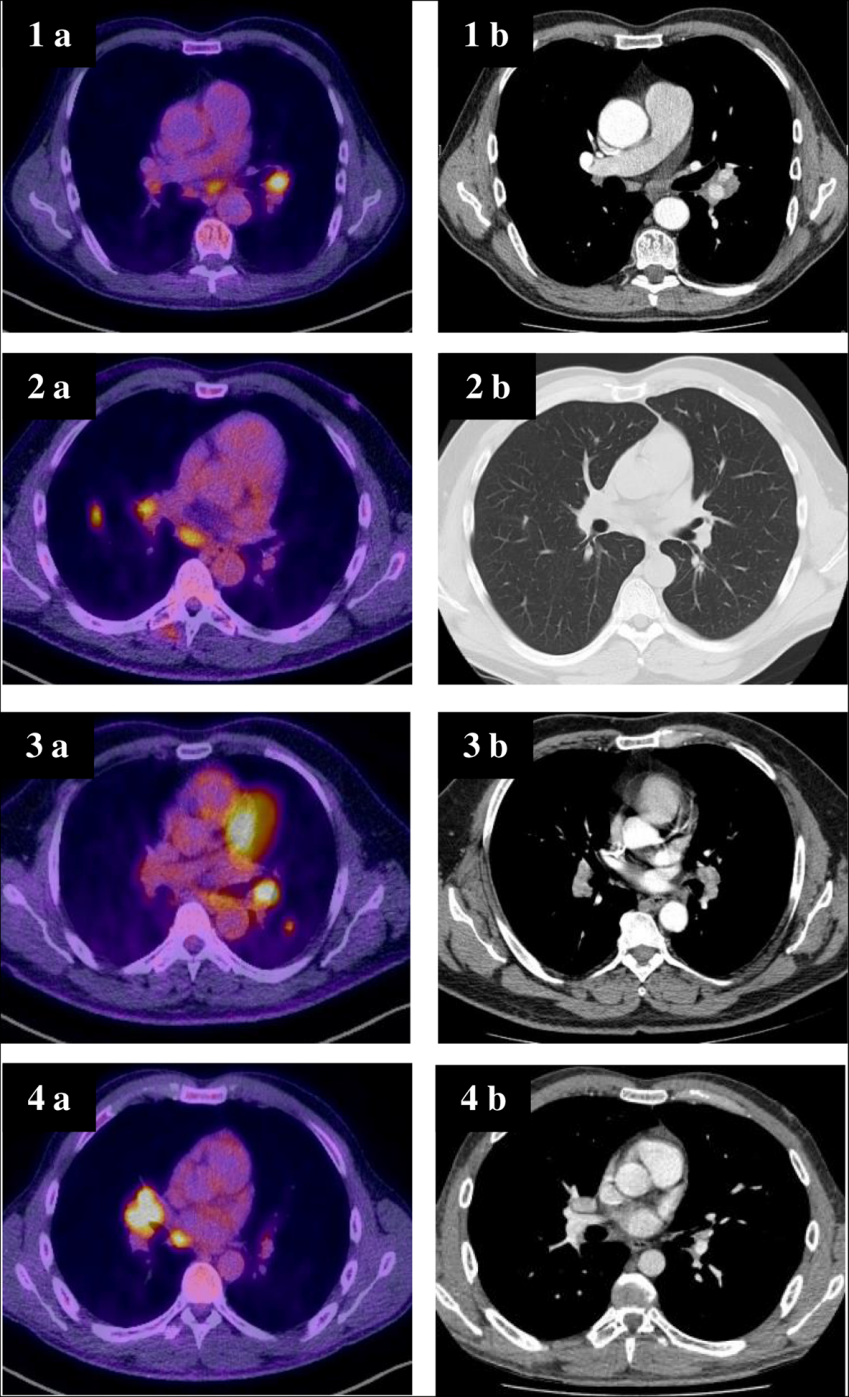
**PET-CT and CT scans, respectively, of four patients suffering from pulmonary tularemia (initial presentation (a) and after completed antibiotic treatment (b)).**

**Figure 2:**
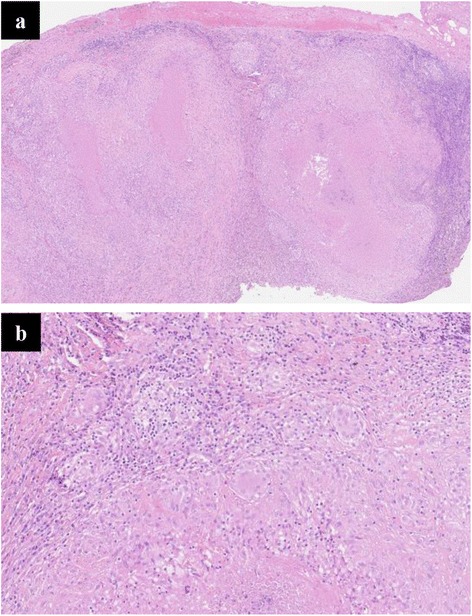
**Hilar lymph node from patient No 1 demonstrating necrotic granuloma (hematoxylin eosin 10× (a) and 20× (b)).**

A 62 year old otherwise healthy pastor was referred to our institution due to dry cough, weight loss of 7 kilograms (9% of body weight) and night sweats. He was a lifelong non-smoker. The symptoms had lasted for about eight weeks. The chest x-ray showed an enlarged left hilus. Computed tomographic (CT) scan of the chest showed a hilar mass of three centimeters with enlarged hilar and mediastinal lymphnodes and a pulmonary nodule of 7 millimeters in the lower left lobe. All of these lesions were highly avid for Fludeoxyglucose in the consecutive positron emission tomography CT fusion scan (FDG-PET-CT, Figure 1) and no other pathological findings were seen. Bronchoscopy failed to reveal any endobronchial abnormality. However, with the aid of endobronchial ultrasonography (EBUS) we were able to identify the hilar mass and to perform EBUS guided transbronchial fine needle aspiration (TBNA) of this lesion. The aspiration revealed normal lymphocytes, necrosis and aggregations of epitheloid cells. No malignant cells were seen. Due to high suspicion of malignancy, thoracoscopic hilar lymphadenectomy was performed. Microscopic examination of the biopsy specimen showed necrotic granuloma (Figure 2). Staining of the biopsy for mycobacteria was negative. In a next step, serological testing for Bartonella henselae, Brucella and Francisella tularensis revealed a titer of 1:320 for Francisella tularensis, whereas the others were negative. Nested PCR was performed on the biopsy specimen for Francisella tularensis and this too was positive. Although the patient was asymptomatic at this time, we established an antibiotic treatment with doxycyclin for the duration of three weeks. After this period, the patient returned to our outpatient clinic with fever and night sweats. This presentation was highly suggestive of a recurrence of an infection with Francisella tularensis. After an antibiotic therapy with garamycin for one week and ciprofloxacin for additional two weeks, the patient's condition improved rapidly. The subsequent course of the patient was uneventful for a follow up period of 27 months. A CT scan performed after the second antibiotic treatment showed a smaller residual lesion (Table 1).

**Table 1:** **Findings, treatment and clinical course of four patients suffering from pulmonary tularemia**

**Patient**	**No 1**	**No 2**	**No 3**	**No 4**
**Lead symptoms**	**Cough, weight loss, night sweat**	**Fatigue, head ace, fever**	**Weight loss, head ace, fever**	**Cough, fever**
Date of diagnosis	Jan 12	Sep 12	Oct 2012	Oct 2012
Duration of symptoms before/after start of therapy (weeks)	14/NA (spontanous recovery)	12/6	9/12	2/2
Agglutination titer for F. tularensis	1:320	1:5120	1:320	1:640
White-cell count (per mm3)/CRP (mg/liter) at first presentation in our institution	4.2/6	11.4/87	5.1/83	6/<3
Nested PCR tissue for F. tularensis	positive	positive	not done	positive
EBUS-TBNA	necrosis, epithelioid cell aggregation	necrosis, epithelioid cell aggregation	necrosis, epithelioid cell aggregation	normal lymphocytes
Surgical biopsy	yes	not done	not done	yes
Probable vector	rabbit feces	tick	unknown	unknown
Treatment	1.doxycyclin	ciprofloxacin	ciprofloxacin	ciprofloxacin
	2.garamycin/cipro-floxacin			
Course	recurrence after doxycyclin	remission	remission	remission
Length of follow up (months)	27	18	16	16
Initial CT scan findings	Hilar mass, enlarged lymph nodes, pulmonary nodule	Pulmonary nodules, enlarged lymph nodes	Hilar mass, enlarged lymph nodes	Mass in the middle, enlarged lymph nodes
CT scan findings after completed treatment	Residual lesion	Small residual parenchymal scar	unchanged	Small residual hilar scar

CRP: C-reactive protein, EBUS-TBNA: endobronchial ultrasound guided transbronchial needle aspiration, CT: computed tomography, PCR: polymerase chain reaction; NA: not applicable.

### Patient No 2

A 49 year old forest official had been well until six weeks before presentation, when fever, intermittent headache and fatigue developed. A chest x-ray and subsequent CT scan revealed multiple nodules in the lower right lobe and enlarged hilar and mediastinal lymphnodes. The patient was referred to our institution for further diagnostic work-up. Bronchoscopy revealed distinct signs of acute bronchitis and an EBUS guided TBNA of the enlarged subcarinal lymph node was done. The aspirate showed necrosis and epithelioid cell aggregations. Since the intrathoracic lesions were highly suggestive of malignancy, a PET-CT scan was performed. All visible pulmonary nodules - as well as the hilar and subcarinal lymph nodes - were highly avid for FDG. Since the aspirate of a repeated TBNA of the subcarinal lymphnode again revealed lymphocytes and epitheloid cell aggregates, we decided to send the patient for diagnostic mediastinoscopy. However, considering the previous experience with patient number one, we additionally sent the patient's serum to be analyzed for Francisella agglutination titer, which was highly elevated (Table 1). Subsequently, we cancelled mediastinoscopy and sent aspirate from the TBNA of the subcarinal lymphnode to perform nested PCR for Franzisella tularensis. This proved to be positive. Antibiotic therapy with ciprofloxacin was established for three weeks and the patient recovered slowly over a time period of about six weeks. A follow up CT scan of the thorax two months after termination of the antibiotic treatment, documented unremarkable findings, apart from a small scar in the parenchyma of the lower right lobe (Figure 1).

### Patient No 3

A 52 year old businessman was referred by his general practitioner due to weight loss of about 15% of body weight, headache and fever. The patient had a 25 pack year smoking history. The symptoms started three weeks before referral. On examination, the temperature was 39.5°C and a pleural rub was heard over the left side of the chest. A chest x-ray and a consecutive CT scan revealed a left hilar mass with central necrosis and multiple enlarged hilar and mediastinal lymph nodes. A bronchoscopy was performed. Apart from signs of an acute bronchitis, the investigation was macroscopically normal. EBUS guided TBNA of an enlarged left-sided hilar lymph node was performed. The aspirate showed lymphocytes and was negative for malignant cells and for PCR for M. tuberculosis. Blood cultures and cultures of bronchial aspiration fluid remained sterile. After bronchoscopy, the general condition of the patient deteriorated further and empiric antibiotic treatment with ceftriaxone was started. Due to a subsequent rash which developed within two days of beginning the ceftriaxone, the antibiotic treatment was changed empirically to levofloxacin. Since the general condition of the patient improved and the fever disappeared, the antibiotic therapy was stopped after ten days. The patient returned home and was advised to see his general practitioner for follow-up consultations, particularly to perform an X-ray of the chest after recovery.

After six weeks, the patient was reassigned due to recurrent fever, night sweats and fatigue. Due to suspicion of lung cancer, a PET-CT scan was performed. The left hilar mass had increased in size and the hilar and mediastinal lymphadenopathies were unchanged when compared to the former CT scan. The lesions were highly FDG avid (Figure 1). We decided to repeat EBUS guided TBNA of the left hilar lymph node and the aspirate showed necrosis and epitheloid cell aggregations. Since PCR for M. tuberculosis and the culture results obtained from the former aspirate were negative, tuberculosis was considered unlikely. Serum of the patient was then sent to be analyzed for Francisella agglutination titer, which was highly elevated (Table 1). Antibiotic treatment with ciprofloxacin was established and the condition of the patient improved slowly. A CT scan three months after stopping the antibiotic treatment was unchanged when compared to the PET-CT scan. By that time, his neutrophil count had recovered and he was feeling well - as he was sixteen months later.

### Patient No 4

A 40 years old topographer had been suffering from fever and a cough that had lasted for two weeks, when he was treated with clarithromycin by his general practitioner. Due to persistently elevated leucocytes, the antibiotic treatment then was changed to cefpodoxime. Although his general condition improved and the elevated leucocytes subsequently became normal, a chest x-ray showed a right hilar mass. A CT scan revealed a 5cm mass in the middle lobe and an enlarged subcarinal lymph node. The patient was referred for further evaluation of suspected lung cancer. EBUS guided TBNA of the enlarged subcarinal lymph node showed normal lymphocytes. A later PET-CT scan revealed a highly FDG avid lesion of the middle lobe, as well as a highly positive subcarinal lymph node (Figure 1). No other lesions were detectable. We decided to repeat EBUS guided TBNA of the subcarinal lymph node in addition to an EBUS guided TBNA of the pulmonary mass. In both aspirates normal lymphocytes were found. A thoracoscopic excision of an enlarged hilar lymph node revealed necrotizing granulomas. Polymerase chain reaction for M. tuberculosis of the tissue was negative, but Francisella agglutination titer in the serum of the patient was markedly elevated. Later on the PCR of the lymph node for F. tularensis was positive (Table 1). Though in the meantime the symptoms disappeared completely, we administered an antibiotic treatment with ciprofloxacin for three weeks. The CT scan of the chest that was performed eight weeks later showed normal findings, except for a small hilar scar (Figure 1).

## Conclusions

Francisella tularensis is a pathogenic species of Gram-negative bacteria. It is a fastidious, facultative intracellular bacterium that is spread by aerosol with high virulence. Depending on the side of infection, tularaemia may present with several clinical manifestations, such as oropharyngeal, ulceroglandular, occuloglandular or typhoidal type. Tularaemia is caused by contact with infected animals manly such as rabbits and rodents. In this case series, the clinical manifestations were nonspecific and, in contrast to the majority of the reported cases, they were remarkably long lasting, both before and after initiation of antibiotic therapy (Table 1).

To the best of our knowledge, our four cases are the first described in the literature where PET-CT-scans are available. Several findings we observed with this technique are remarkable. Firstly, in all four cases we did not detect any other manifestation in the body except the lesions in the lung and the hilar and mediastinal lymph nodes, respectively. In our opinion, this fact strongly argues for a primary affection of the lungs as a result of inhalation of the bacteria. Therefore, in our cases, hematogeneic spread causing secondary pulmonary involvement seems unlikely. Secondly, in all cases, the result of the PET-CT scan - much like the result of the CT scan - was indistinguishable from malignancy, particularly lung cancer. The latter phenomenon has already been published in an earlier study [6]. The impressive PET-CT findings of the current cases underline the necessity of intransigent and rigorous work up for cancer diagnosis and cancer staging even in cases with supposed highly typical PET-CT findings. Thirdly, in combination with EBUS guided TBNA, a PET-CT scan performed before bronchoscopy might be helpful in defining the most promising target lesion in order to reach the highest possible diagnostic yield. We believe that representative and typical findings in EBUS guided TBNA aspirates - in combination with positive serologic testing - are sufficient to establish the diagnosis of pulmonary tularaemia and to start antibiotic treatment. Nevertheless, in our opinion, two to three months after finishing the antibiotic treatment, a follow up CT scan is mandatory in this form of tularaemia in order not to miss malignancy. Considering the rarity of pulmonary tularaemia, the severity of consequences and the paucity of scientific data, this costly approach seems justifiable.

The follow up CT scans of the current cases were interesting in several ways. In two of the four cases beside a small scar all changes disappeared subsequently whereas in two cases the findings disappeared only partially and remained mainly unchanged, respectively. Interestingly, this latter particular patient (No 3) suffered the longest from persistent symptoms after initiation of antibiotic treatment. Both observations might be the consequences of persistent inflammatory reaction.

We did not find a common source of infection in the four cases described here.

## Consent

Written, informed consent was obtained from the four patients for publication of this case report and any accompanying images. A copy of the written consent is available for review by the editor of this journal.

## Abbreviations

CT: Computed tomography
CRP: C-reactive protein
EBUS: Endobronchial ultrasonography
FDG: Fludeoxyglucose
NA: Not applicable
PCR: Polymerase chain reaction
PET-CT scan: Positron emission tomography computed tomography fusion scan
TBNA: Transbronchial fine needle aspiration
